# Nutritional composition, heavy metal content and *in vitro* effect on the human gut microbiota of *Talitrus saltator*, an underutilized crustacean from the Atlantic coast

**DOI:** 10.3389/fnut.2022.943133

**Published:** 2022-10-13

**Authors:** Aroa Lopez-Santamarina, Alejandra Cardelle-Cobas, Alexandre Lamas, Alicia Mondragon-Portocarrero, Alberto Cepeda, Jose Manuel Miranda

**Affiliations:** Laboratorio de Higiene Inspección y Control de Alimentos, Departamento de Química Analítica, Nutrición y Bromatología, Universidade de Santiago de Compostela, Lugo, Spain

**Keywords:** gut microbiota, chitin, crustacean, *Tallitrus saltator*, Proteobacteria, *Bifidobacterium*

## Abstract

In this study, an undervalued marine crustacean (*Talitrus saltator*) was characterized in terms of nutritional and heavy metal composition and its potential to affect human gut microbiota. Nutritional analysis of this crustacean revealed that it complies with the criteria established in European legislation to include nutritional claims in their labeling, such as “source of fiber,” “low in fat,” “low in sugars” and “high in protein.” The analysis of the heavy metal content did not reveal any risk derived from the presence of Cd, Hg, or Pb, whereas essential metals contained in 100 g exceeded the minimum daily requirements recommended in Europe for Zn (19.78 mg/kg), Cu (2.28 mg/kg), and Fe (32.96 mg/kg). Using an *in vitro* system, the effect of *T. saltator* on the human colonic microbiota shows some beneficial effects, such as fermentation-maintained populations of *Bifidobacterium* or *Lactobacillus*, did not increase Firmicutes *phylum* counts, decreased the Firmicutes/Bacteroidetes ratio, and stimulated 11 metabolic pathways with respect to baseline. These results are unusual in a high protein content-food. However, negative effects were also found in gut microbiota relative proportions, such as an increase in the Proteobacteria *phylum* and especially some opportunistic bacteria from this *phylum*, probably due to the antimicrobial effect of chitin on other groups more sensitive to its effect. This work shows for the first time the effect of *T. saltator* on human colonic microbiota using and *in vitro* system. The presence of chitin in its composition could provide some beneficial effects by modulating the microbiota, but as *T. saltator* is a high-protein food, more studies should be carried out showing these benefits.

## Introduction

According to demographic forecasts, it is expected that by 2050, there will be ten billion people in the world (1). As a result, food production in 2050 will need to double with respect to current production to feed the entire world population. Consequently, it will be necessary to make better use of the different food sources (2).

One alternative sustainable food production system proposed to prevent future food shortages is to increase the consumption of other animal foods, such as insects, a good source of proteins that also contain chitin. Chitin is a modified polysaccharide (poly-beta-1,4-N-acetylglucosamine) considered an insoluble fiber with potential prebiotic properties that could benefit human health (3, 4). Chitin is the primary component of the exoskeleton, respiratory linings, digestive, and excretory systems of arthropods (3) and can also be found in other organisms, such as fungi, crustaceans, mollusks, protozoa, and green algae (4). In fact, after cellulose, chitin is the most abundant biomass on Earth (5). Chitin and its derivative chitosan have a wide range of biological activities, such as antioxidant or antibacterial effects (6). Consumption of chitin has been shown to contribute to the improvement of human health in many aspects, such as improving glucose intolerance, increasing insulin secretion, and relieving dyslipidemia, as well as antiviral, anticancer and antifungal activities (5, 7). In recent years, scientific and industrial interest in chitin has increased due to its biodegradability by some colon bacteria, as well as its biocompatibility, low toxicity, and antimutagenic properties (4), suggesting that its regular inclusion in the human diet could provide health benefits by means of a selective activity on human gut microbiota (8).

Although human consumption of insects (entomophagy) is gaining traction in geographical areas that traditionally did not practice entomophagy, such as North America and Europe (3), there are still many people who are repulsed by this dietary practice (9). Thus, one alternative source of chitin is discarded crustaceans because most people from Western countries have positive perceptions regarding seafoods (10).

The gut microbiota (GM) is the name for the microbe population living in the gastrointestinal tract, particularly in the colon. The main phyla found in the human gut microbiota are Firmicutes and Bacteroidetes, and in lower proportions Proteobacteria and Actinobacteria. GM composition and activity are of major importance for the health and wellbeing of the individual or even in the maintenance of immunological activity (11).

*Talitrus saltator* is a semiterrestrial amphipod crustacean of the *Talitridae* that usually lives in the supralittoral zone of the beaches of the Northeast Atlantic and the Mediterranean Sea (12). Their diet is basically centered on the detritus of animals, plants, and algae that they find when foraging (13). *T. saltator* integument consists of an outer non-cellular structure (cuticle), a middle cellular layer (epidermis), and an inner unicellular layer (basement membrane). The cuticle is mainly composed of two layers: the epicuticle, which lacks chitin, and the procuticle, which is the layer of most interest due to its chitin content (14).

In this work, *T. saltator*, a currently underutilized crustacean species from the Atlantic coast of Spain, was proximate, and its heavy metal contents were characterized. Afterward, the effects of *T. saltator* consumption on the human GM were investigated using an *in vitro* system that simulates the human colon. To the best of our knowledge, this is the first time that *T. saltator* was nutritionally characterized and its content in heavy metals, and its effect on human GM was determined.

## Materials and methods

### Crustacean sampling

*T. saltator* were collected from the coast of Ribeira (A Coruña, Spain). After collection, they were washed with seawater to remove sand, algae, and other detritus and then transferred to an ice chest in the laboratory for immediate processing. *T. saltator* were crushed and freeze-dried prior to testing. The freeze-drying process was carried out by using a vacuum freeze drier (Labconco™ 77560-LYPM-LOCK6) (Kingston, NY, USA) under a vacuum pressure ≤ 140 × 10^–3^ Mbar and a condenser temperature of –46°C. Subsequently, the samples were stored in the dark at room temperature until use within 3 days.

### Proximate and mineral composition of *Talitrus saltator*

In the absence of previous studies on the nutritional value of *T. saltator*, proximate analysis was carried out, in duplicated, in our laboratory. The nutritional analysis was carried out following official methodologies established by the Official Association of Analytical Chemists (15). Moisture content was determined by drying in a porcelain capsule at 100–105°C until constant weight. Protein content was determined by nitrogen content determination, fat was determined by petroleum ether extraction, dietary fiber was determined by the enzymatic-gravimetric method *via* the Megazyme^®^ total dietary fiber assay kit (Megazyme, Wicklow, Ireland), and ash content was determined by muffle furnace ashing at 500°C. The sodium content was determined by atomic absorption. Carbohydrate levels and caloric content were determined through calculations.

Regarding minerals, *T. saltator* was analyzed for Cd, Cu, Hg, Fe, Pb, and Zn by inductively coupled plasma–mass spectrometry (ICP–MS) using an Agilent 7,700 × spectrometer (Agilent Technologies, Santa Clara, CA, USA). The Hg concentration was measured by the Hg cold vapor flow injection technique. The samples were digested using a 3:1 solution of nitric acid (HNO_3_) and hydrogen peroxide (H_2_O_2_) (Merck, Darmstadt, Germany). Then, 250 mg of freeze-dried sample was added to a closed microwave accelerated digestion system raised from 30 to 90°C for 10 min and maintained for 20 min. The final digested sample solution was diluted to 25 ml with deionized water and analyzed for metals (16). Sample blanks were prepared in the laboratory in a similar manner to real samples but without crustaceans. Chitin content in *T. saltator* samples was determined by absorbance using a V-630 Jasco (Madrid, Spain) spectrophotometer following a method reported in the literature (17).

### *In vitro* simulation of oral, gastric, and small intestinal digestion

To determine the effects of *T. saltator* ingestion on human GM, *in vitro* digestion of *T. saltator* was carried out by simulating the human gastrointestinal tract following the INFOGEST consensus method (18). A 10 g sample of freeze-dried *T. saltator* was used for each replicate (*n* = 3).

After the *in vitro* digestion process, the beakers with the *T. saltator* solution were placed in the cooling chamber for 15 min at 4°C. After that, small intestinal absorption was simulated by dialysis (molecular weight cutoff of 1,000 Da, Spectra/Por^®^, Waltham, MA, USA) against distilled water for 2 days with agitation and then frozen for subsequent freeze-drying. All *in vitro* digestion processes, including dialysis, were performed in triplicate.

### Volunteers and preparation of stool samples

Stool samples were obtained from three healthy human volunteers (one male and two females, 32–50 years old) who participated in a clinical trial authorized by the Galician Bioethics Committee (trial 270/2018). These volunteers did not ingest antibiotics or pharmaceutical preparations of pre/pro/postbiotics in the 6 months prior to sample collection and without any gastrointestinal disorder.

Stool samples (between 10 and 30 g each) were collected by volunteers in sterile containers and given to the laboratory within 2 h of collection. Once received, the stool samples were diluted 1:10 with phosphate-buffered saline (PBS; 0.1 M, pH 7.0) (19) and then homogenized (MIX2, AES, France) for 5 min. The diluted feces were stored in sterile jars and frozen at –20°C until use.

### *In vitro* simulation of human colonic fermentation

The *in vitro* human colonic simulation was performed according to Cardelle-Cobas et al. (19). Briefly, a sterilized fermentation vessel containing a basal culture medium without any source of carbon was used to simulate human distal colonic fermentation. In addition to the *T. saltator* fermentation, a trial without a carbon source (negative control) was performed for each voluntary assay (*n* = 3).

The conditions of the human distal colon were simulated by adjusting different parameters. An anaerobic atmosphere was achieved by a continuous supply of pure grade N_2_ (Nippon Gases, Madrid, Spain) through a 0.2 μm polytetrafluorethylene filter (Sartorius Stedim Biotech GmbH, Gottingen, Germany). A thermostatic bath (Pharmacia Biotech, Amsterdam, Netherlands) was used to simulate the human internal body temperature by continuously recirculating water at 37°C throughout the vessel’s water jackets. The pH was adjusted at 6.8, as in eubiotic colonic pH, controlled with a pH regulator (Hanna Instruments, Eibar, Spain), by means of 1 M NaOH or HCl addition, as appropriate.

All chemical compounds used were obtained from Sigma–Aldrich, Merck (Darmstadt, Germany), or Panreac (Barcelona, Spain). Each fermentation vessel was filled, under aseptic conditions, with 200 mL of autoclaved nutrient basal medium (19), adjusted to pH 6.8 and left overnight with a stream of O_2_-free N_2_ with stirring. Next, the sterilized substrate of *T. saltator* was dissolved in 52 mL of the same autoclaved medium and added to the vessels at a final concentration of 1% (w/v). Finally, the vessels were inoculated with 10% (v/v) (28 mL) of the previously prepared diluted feces.

The aliquot samples (3 mL) were removed from each vessel after 0, 10, 24, and 48 h of fermentation for bacterial DNA extraction, which was used for both real-time polymerase chain reaction (*q*PCR) analysis and 16S ribosomal RNA (rRNA) amplicon sequencing.

### Bacterial DNA extraction from fermentation samples

Bacterial DNA was extracted from the fermentation samples by using the DNA Realpure Spin Food-Stool Kit^®^ (Real, Durviz S. L, Valencia, Spain) following the guidelines established by the manufacturer for fecal samples. A total of 1.2 mL of sample (fermentation vessels) was centrifuged at 6,100 *g* to obtain a pellet that was recovered and used for DNA extraction. Extracted DNA was then quantified using a Qubit™4 fluorometer (Invitrogen, Thermo Fisher Scientific, Carlsbad, CA, USA) and the DNA HS Assay Kit (Invitrogen, Thermo Fisher Scientific, Eugene, OR, USA). After quantification, DNA samples were freeze stored at –20°C until further analysis.

### Bacterial population quantification by *q*PCR

*q*PCR assays were carried out in duplicate (*n* = 2) for each assay carried out with a volunteer (*n* = 3) to characterize fecal bacteria using *phylum*- and species-specific primers for total bacteria, Firmicutes, Bacteroidetes, Actinobacteria, Proteobacteria, *Lactobacillus*, and *Bifidobacterium*, based on López-Santamarina et al. (20). Briefly, *q*PCR experiments were performed in QuantStudio 12K Flex (Applied Biosystems, Life Technologies Holding, Singapore, Singapore) equipment using fast SYBR™ green master mix (Applied Biosystems, Vilnius, Lithuania). All PCR determinations were carried out in triplicate using a total volume of 10 μL of reaction containing 1 μL of each sample DNA, primers (0.4 μL) added at a concentration of 200 nM (for each primer), 5 μL of fast SYBR™ green master mix (Applied Biosystems), and 3.2 μL of molecular biology grade water. The thermal cycling conditions consisted of an initial DNA denaturation at 95°C for 10 min, followed by 45 cycles of denaturation at 95°C for 10 s, primer annealing at an optimal temperature for 20 s, and a final extension at 72°C for 15 s. Finally, melt curve analysis was performed by slowly cooling the reactions from 95 to 60°C (0.05°C per cycle) with simultaneous measurement of the SYBR green signal intensity. Melting-point determination analysis allowed confirmation of the specificity of the amplification products.

The bacterial counts measured in copies/mL were calculated by comparing against the threshold cycle (Ct) values obtained from the standard curves with Quant Studio 12K Flex Software (Applied Biosystems). Standard curves were constructed for each experiment by using 10-fold serial dilutions of bacterial genomic DNA (of known concentration) from pure cultures, corresponding to 10^1^–10^10^ copies/mL.

The pure cultures used to construct the standard curves were obtained from different collections of type cultures: The Spanish Collection (CECT), the Belgian Coordinated Collections of Microorganism (LMG), and the German Collection of Microorganisms and Cell Cultures GmbH (DSM)—as follows: *Enterobacter cloacae* CECT 194, *Clostridium perfringens* CECT 376, *Bifidobacterium longum* CECT 4503, *Bacteroides vulgatus* LMG 17767, and *Lactobacillus reuteri* DSM 20016. Each bacterial strain was grown in its recommended culture media and growth conditions. The final data were expressed as an average of the duplicate values obtained in the analyses. The efficiency of the reaction for all pairs of probes was determined by using the slope of the calibration curve obtained for each of the bacterial groups analyzed, namely, E = 10^(–1/slope). The primer pair efficiency ranged from 91% (E = 1.91) to 118% (E = 2.18), with slopes in the range of –3.63 to –2.92.

### 16s ribosomal RNA amplicon sequencing

For 16S rRNA amplicon sequencing, similar methods were followed to those previously reported (20). Twelve microliters of DNA extracted from each sample was used to perform the libraries, and the Ion GeneStudio™ S5 System (Life Technologies, Carlsbad, CA, USA) was used. The 16S hypervariable regions were amplified with two sets of primers, v2-4-8 and v3-6,7-9, and libraries were then constructed by using the Ion 16S™ Metagenomics Kit (Life Technologies) and the Ion Xpress™ Plus Fragment Library Kit (Life Technologies). Libraries containing equal amounts of PCR products pooled with a barcode were prepared by using the Ion Xpress™ Barcode Adapters Kit (Life Technologies). Then, these libraries were quantified by using the Ion Universal Library Quantitation Kit (Life Technologies). Next, 10 pM of each library was pooled and loaded on an Ion OneTouch™ 2 System (Life Technologies), which automatically performs template preparation and enrichment. Template-positive ion sphere particles were enriched with Dynabeads™ MyOne™ Streptavidin C1 magnetic beads (Invitrogen, Carlsbad, CA, USA) by using an Ion One Touch ES instrument. Finally, an Ion 520™ chip (Life Technologies) was loaded with the samples on an Ion GeneStudio™ S5 System sequencer using the Ion 520™ and Ion 530™ Loading Reagents supplied in the OT2-Kit (Life Technologies).

### Statistical and bioinformatic analysis

The results of proximate composition and heavy metal content prior to and after digestion simulation as well as the results obtained for *q*PCR analysis were subjected to statistical comparison by analysis of variance (ANOVA) and a *post hoc* Tukey test. SPSS^®^ for Windows (SPSS Inc., Chicago, IL, USA) was used for these analyses. In all cases, differences were considered significant at *P* < 0.05.

For the analysis of 16S rRNA amplicon sequencing, the raw sequencing reads were obtained from Torrent Suite software (v.5.12.2.) as BAM files, which were converted to fastq files with BEDTools wrapped into the Public Galaxy Server (v. 21.05).^1^ The fastq files were processed with QIIME 2 software v. 2021.8 (21). To produce amplicon sequence variants (ASVs), the DADA2 method was used for quality filtration (Q score > 20), trimming, denoising, and dereplication. Samples with features (taxa) with a total abundance (summed across all samples) of < 10 were removed. Then, ASVs were aligned with mafft and used to construct a phylogenetic tree with fasttree. α*-* and β*-*diversity metrics were estimated by using q2-diversity core-metrics-phylogenetic after samples were rarefied to a sequencing depth of 49,000 reads. Taxonomy was assigned to ASVs by using the q2-feature-classifier classify-sklearn naïve Bayes taxonomy classifier against the Greengenes 13_8 99% operational taxonomic unit (OTU) reference sequences. The PICRUSt online Galaxy version on the Huttenhower Lab (v1.0.0) server was used to predict the metagenome functional content from marker gene surveys and full genomes. Functional metagenomes were categorized based on the Kyoto Encyclopedia of Genes and Genomes (KEGG) pathway database at hierarchy level 3.

STAMP software (v 2.1.3) for the “Statistical Analysis of Taxonomic and Functional Profiles” (22) was used to determine differences in metabolic functions. Welch’s *t*-tests with Bonferroni correction were used to determine significant differences in the relative abundance of 20 selected KEGG pathways. In addition, differences in the relative abundance of the most common species were determined by using a G-test (with Yates’ correction) + Fisher’s exact test with Bonferroni correction.

## Results and discussion

### Nutritional and heavy metal composition

The proximate and heavy metal compositions of *T. saltator* are shown in Table 1. The nutritional composition is close to another discarded crustacean typical of the Galician coasts, such as the crayfish (*Munida* spp.) (23), which showed a dry matter content of 21.38 g/100 g and a fat content of 0.9 g/100 g. The fat content (2.2 g/100 g) was more similar to those obtained for other types of discarded crustaceans used to produce crab substitutes (24), whose fat content ranged between 2 and 3%. According to the results obtained in the present study, the protein/fiber ratio (2.77) is lower than that of some traditional fiber sources, such as wheat (4.7), and close to that of other cereals, such as oats (2.05) (25), and that of some insect meals, such as grasshoppers (3.3) or black soldier fly larvae (2,42) (26), the relationship between protein and fiber content is important for GM due to the known dysbiotic effect caused by the ingestion of high-protein foods (4). The nutritional composition of *T. saltator* would allow its valorization through different nutrition claims, as established in European Regulations (27). Chitin content was 52.5 ± 4.5 mg/100 g, lower than those usually obtained from other crustaceans shells, although it should be taken into account that in the current work chitin content are referred to the whole crustacean an not only for their shells (17). According to its proximate composition, *T. saltator* could be marketed under the claims “low fat content” (< 3 g of fat/100 g product), “low sugar content” (< 5 g sugar/100 g product), “source of fiber” (< 1.5 g of fiber/100 g product), and “high protein content” (> 20% of the energy content of the product is provided by protein). The nutrition claims approved by European Regulation (27) are a factor that favorably conditions the purchase of food in those consumers who identify and select products with healthier ingredients (28).

**TABLE 1:** Nutritional composition and mineral content of *Talitrus saltator*, raw and after upper *in vitro* digestion.

	Raw *T. saltator*	*T. saltator* after upper *in vitro* digestion
**Nutritional composition**		
Dry extract^(1)^	20.84^b^ ± 0.05	37.50^a^ ± 0.04
Moisture^(1)^	79.16 ± 0.01	62.50 ± 0.06
Fat^(1)^	2.23^b^ ± 0.32	5.28^a^ ± 1.16
Protein^(1)^	7.70^a^ ± 0.11	5.09^b^ ± 0.01
Carbohydrates^(1)^	3.40^b^ ± 0.25	11.18^a^ ± 1.12
Dietary fiber^(1)^	3.25^b^ ± 0.60	10.65^a^ ± 0.06
Sodium^(1)^	0.21^a^ ± 0.11	0.11^b^ ± 0.01
Ash^(1)^	7.50^b^ ± 1.52	15.95^a^ ± 0.33
Caloric content^(2)^	57.97^b^ ± 0.05	91.30^a^ ± 1.56
Chitin content^(4)^	52.50 ± 4.50	172.20 ± 5.50
**Metals**		
Cd^(3)^	0.48^b^ ± 0.07	2.43^a^ ± 0.52
Cu^(3)^	2.28^b^ ± 0.06	8.32^a^ ± 0.21
Hg^(3)^	0.21^b^ ± 0.03	1.34^a^ ± 0.14
Fe^(3)^	32.96^b^ ± 1.25	139.18^a^ ± 7.21
Pb^(3)^	0.07^b^ ± 0.02	0.23^a^ ± 0.12
Zn^(3)^	19.78^b^ ± 0.10	115.52^a^ ± 0.53

^(1)^g/100 g; ^(2)^Kcalories/100 g; ^(3)^mg/kg; ^(4)^mg/100 g.

^a,b^Different letters indicate significant differences between rows (*P* < 0.05).

Heavy metals are currently considered chemical hazards by community legislation, and their content must therefore be controlled in foodstuffs intended for human consumption. The contamination of fishery products by heavy metals is highly dependent on human activity, as sources of contamination include oil spills, anchoring of ships and discharge of domestic waste, agricultural runoff, industrial discharges, and heavy waste (29).

In this regard, European Regulations set maximum levels for fresh crustaceans for some heavy metals, such as Cd (1 mg/kg maximum level *vs*. 0.48 mg/kg *T. saltator*), Hg (0.5 mg/kg maximum level *vs*. 0.21 mg/kg *T. saltator*) or Pb (1.5 mg/kg maximum level *vs*. 0.07 mg/kg *T. saltator*) (30). Hg is well known for its toxic effects at the neuronal level, especially in young individuals, while Cd and Pb have been shown to cause cell damage and reduced reproductive and growth rates and death (31). For all cases, the levels were below the reference values established by European laws (30).

Not all heavy metals pose a direct risk to human health. Elements such as Fe, Ca, Cu, and Zn are required for the growth, enzymatic reactions, and metabolic activities of marine organisms (32). Moreover, GM requires certain heavy metals, such as Fe, Cu, or Zn, for their growth and metabolism (33). Zn is a trace element whose intake by humans is traditionally less deficient in several parts of the world. To date, 16% of the world’s population is estimated to be zinc-deficient (34), and its deficiency is associated with several common diseases, critically impairs the immune system, and increases the risk for infection and disease-related mortality (34). Thus, 100 g of *T. saltator* would provide 198% of the minimum daily Zn requirement for adults, as stipulated in Council Directive 2008/100 (35). In the case of Cu and Fe, *T. saltator* has also proven to be an excellent source, with 100 g providing 288% of Cu and 235% of Fe of the minimum daily requirements (35).

In all investigated metals, there was a significant increase in the metal content of *T. saltator* after digestion in the upper intestinal tract. The proportion of nutrients vary after digestion because some components increase and other decrease after the process with enzymes and acidic conditions, however the use of membranes of dialysis also possess limitation when using in assays of simulation of small intestine absorption or bioavailavility (20, 36). Complexes between the different macromolecules of *T. saltator*, especially protein can be formed with metals and It is the case of Zn complexes, for example, that cannot cross the dialysis membranes when the pore is too small a factor relevant to the present work given that the dialysis membrane has a 1 kDa cutoff. There is a similar situation for Fe: this metal is part of high-molecular-weight complexes such as ferritin and cannot cross the dialysis membranes, although humans absorb iron at the intestinal level.

### qPCR analysis

The results of *in vitro* fermentations showed that for most of the bacterial groups investigated, the ingestion of whole *T. saltator* stimulated their growth, indicating that they have been metabolized and fermented by the colonic microbiota (Table 2). Most effects were found after the first 10 h of fermentation, and the remaining bacterial groups were stable in most cases in the period of 10–48 h of fermentation, with the only exception of the Bacteroidetes *phylum* in the *T. saltator*-added samples. As reported previously, the Firmicutes and Bacteroidetes *phlylum* were predominant in the fecal samples, as usual in Western-country individuals (4, 37). Firmicutes showed an increase in negative controls during the 48-h period of fermentation, while in *T. saltator* added samples, Firmicutes counts remained stable. Bacteroidetes counts decreased when *T. saltator* was added, recovering and even exceeding the initial counts and counts found for negative controls at 48 h of fermentation. These results showed that, despite causing an initial inhibition, *T. saltator* used as substrate by Bacteroidetes promoted its growth. Previous works pointed out that Bacteroidetes have a wide repertoire of genes encoding enzymes involved in polysaccharide metabolism and usually increase their counts when complex carbohydrates are metabolized and fermented (38). Considering both phyla together, the Firmicutes to Bacteroidetes ratio, for which elevated values have been associated with a predisposition to obesity (37), remained at lower values for the samples with *T. saltator* than in negative controls during the entire assay, which is a positive finding.

**TABLE 2:** Bacterial population (log_10_ DNA copies/mL) in the *in vitro* colon model after 0, 10, 24, and 48 h of fermentation.

		*T. saltator*			Negative controls		
	0 h	10 h	24 h	48 h	10 h	24 h	48 h
All bacteria	7.58^b^ ± 0.64	10.23^a,A^ ± 0.32	10.15^a,A^ ± 0.24	10.64^a,A^ ± 0.05	10.66^a,A^ ± 0.01	10.69^a,A^ ± 0.03	10.62,A^a^ ± 0.01
Firmicutes	5.97^b^ ± 0.14	6.10^b,A^ ± 0.16	6.09^b,A^ ± 0.10	5.81^b,A^ ± 0.15	7.21^a,B^ ± 0.01	7.24^a,B^ ± 0.01	7.12^a,B^ ± 0.01
Bacteroidetes	6.19b ± 0.26	5.02^c,A^ ± 0.12	5.39^c,A^ ± 0.29	6.72^a,A^ ± 0.31	6.03^b,B^ ± 0.09	6.00^b,A^ ± 0.01	5.87^b,A^ ± 0.18
Proteobacteria	5.34^b^ ± 0.21	9.44^a,A^ ± 0.25	9.22^a,A^ ± 0.19	9.32^a,A^ ± 0.30	9.77^a,A^ ± 0.02	9.84^a,A^ ± 0.06	9.57^a,A^ ± 0.02
Actinobacteria	5.56^a^ ± 0.08	5.66^a,A^ ± 0.15	5.68^a,A^ ± 0.25	5.27^a,A^ ± 0.11	5.37^a,A^ ± 0.01	4.98^a,b,A^ ± 0.02	4.65^b,A^ ± 0.02
*Bifidobacterium*	4.09^a^ ± 0.14	4.12^a,A^ ± 0.11	4.18^a,A^ ± 0.21	4.07^a,A^ ± 0.14	4.08^a,A^ ± 0.13	3.96^a,A^ ± 0.32	3.52^b,A^ ± 0.11
*Lactobacillus*	1.43^a^ ± 0.29	1.33^a,A^ ± 0.32	1.30^a,A^ ± 0.38	0.97^a,A^ ± 0.31	0.46^b,A^ ± 0.01	0.23^c,A^ ± 0.01	0.01^c,A^ ± 0.01

Different capital letters indicate significant differences (*P* < 0.05) between Talitrus saltator and negative controls at 10, 24, and 48 h. Different lowercase letters indicate significant differences (*P* < 0.05) with time for the same substrates. The results are expressed as the mean ± standard error (*n* = 6).

The Proteobacteria *phylum* abundance increased with respect to the initial counts in both the *T. saltator*-treated samples and the negative controls. In the case of *T. saltator* this increase may be due to the protein and fat content in the substrate. An increased prevalence of Proteobacteria is considered a potential diagnostic signature of dysbiosis and risk of disease (39), usually related to intake of animal-origin fats (38). However many Proteobacteria are commensals and not associated with dysbiosis (40). This increase in genus belonging to the proteobacteria phylum was observed for similar assays carried out with different insect species of edible insects (41). Actinobacteria remained stable in *T. saltator*-added samples, whereas in the case of negative controls, Actinobacteria abundance decreased after 24 h of fermentation. A similar evolution was found for its main genus (*Bifidobacterium*), which did not vary in *T. saltator* samples, but after 24 h of fermentation, it began to decrease in negative controls and for the *Lactobacillus* genus. Both *Bifidobacterium* and *Lactobacillus* are probiotic genera that ferment complex carbohydrates and thus are stimulated by complex intake (41). Similar results were obtained when using *Tenebrio molitor flour* as a substrate for *in vitro* fermentation (11). Recent works have demonstrated that some *Bifidobacterium* and *Lactobacillus* can grow stimulated by peptide or amino acid fermentation (42). Also, this bacteria can be stimulated for the presence of undigestible carbohydrates which can be digested by them as inulin or fructooligosaccharides, in this case some of the carbohydrates present in *T. saltator* seems to be used because this genera remains constant along the fermentation time, whereas in the negative controls, in the absence of carbohydrates and in the presence of proteins, both genera began *to* decrease even after 10 h of fermentation. Similar results for *Lactobacillus* and Bifidobacterium genus where found previously in digested *T. molitor* flour (11).

### Amplicon 16s ribosomal RNA sequencing

**FIGURE 1:**
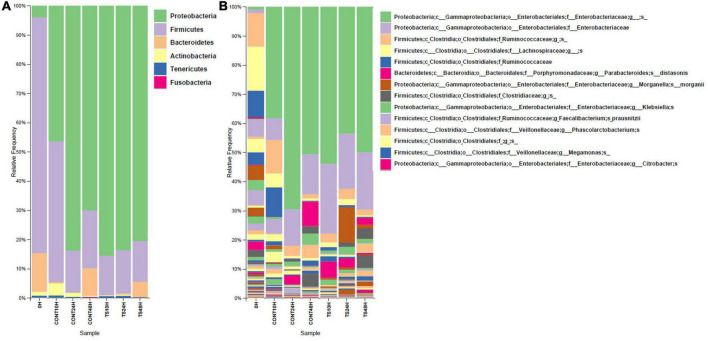
Relative abundance (%) of operational taxonomic units from different bacterial phyla **(A)** and genera **(B)** for group mean values. The X-axis shows different substrates evaluated at different assay times (0, 10, 24, and 48 h). Due to the large number of reported genera, only the top 14 most abundant were placed. These results are the average of the three volunteers on each substrate and at different times. CONT, Negative controls; TS, *T. saltator*.

*In vitro* simulation of the GM is a method to reduce the use of *in vivo* models, which reduces both the use of experimental animals and the potential risks for humans (20). Products of *in vitro* fermentations were used to evaluate GM composition at the relative level for both phyla (Figure 1A) and genera (Figure 1B).

**FIGURE 2:**
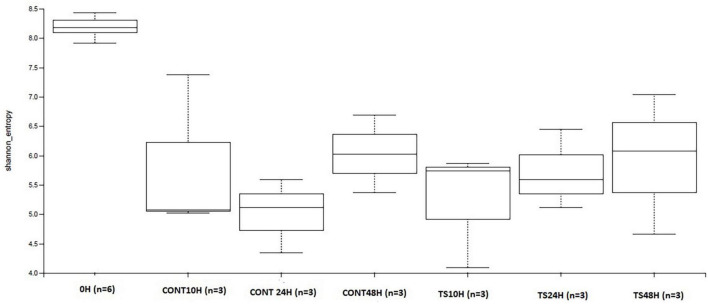
Evolution of α-diversity of operational taxonomic units in *Talitrus saltator*-added samples at 48 h of fermentation with respect to baseline and with respect to negative control samples at 48 h of fermentation. CONT, Negative controls; TS, *T. saltator*.

One of the factors that are often considered an indicator of good gastrointestinal health is that GM should be abundant and diverse, measured by α- and β-diversity. The α-diversity of OTUs measured by Shannon (Figure 2) and Chao-1 indexes showed that initial samples were more diverse than those products of fermentations for both *T. saltator* added samples and negative controls (no source of carbon added). *T. saltator* added-samples and negative controls showed similar α-diversity during the 48 h fermentations process. This is usual because microbial diversity tends to decrease when a single type of substrate is added, favoring those microorganisms better adapted to their fermentation and metabolization to proliferate more than others (20). For b-diversity, no differences among samples were found, so distinction among assays was not observed (data not shown).

With respect to Firmicutes, this *phylum* constitutes the main population in the GM of the subjects participating in the study and was therefore the most relatively abundant *phylum* in the initial samples. A high relative abundance of this *phylum* is associated with diets high in animal protein and fat and constitutes one of the most frequent deviations in the GM of populations from Western countries with respect to those hunter-gatherer populations (37). Considering that *T. saltator* contains approximately 3 times more protein than dietary fiber (7.70 g/100 g vs. 3.40 g/100 g), it might seem that *T. saltator* would be a suitable substrate for the growth of Firmicutes. However, one of the factors that have been attributed to chitin is its ability to interfere with protein absorption as an anti-nutritive agent (43). In fact, the results obtained showed that Firmicutes relative abundance decreased more rapidly for *T. saltator*-added samples than for negative controls, suggesting that the presence of the sample had a higher inhibitory effect on Firmicutes than its absence. This property has been previously published in studies investigating the effects of insect ingestion on GM compared to other protein-rich foods such as meat (44).

The Bacteroidetes *phylum* includes primary degraders of complex polysaccharides, such as *Bacteroides* (5). The results obtained seem to indicate that other components of *T. saltator* samples could be more readily metabolized initially than complex polysaccharides, such as substrates, than can be metabolized by Proteobacteria. Proteobacteria is a *phylum* included within the bacterial groups favored by diets with high fat proportions and also protein. In the present trial, the *phylum* Proteobacteria notably increased after the addition of *T. saltator*, with a maximum increase at 10 h and a slight decrease thereafter (data not shown), but always at levels higher than at the start of the trial. The observed increase may be due to the antibacterial effect of chitin (11), which is more active against other bacterial groups than against Proteobacteria, since this phylum is usually more resistant to the action of antibacterial agents (37, 45).

With respect to the other phyla that were present in the samples investigated (Actinobacteria, Fusobacteria, and Tenericutes), their relative amounts were low, and no clear effect was observed in the samples with *T. saltator* with respect to those of time 0 or the negative controls.

**FIGURE 3:**
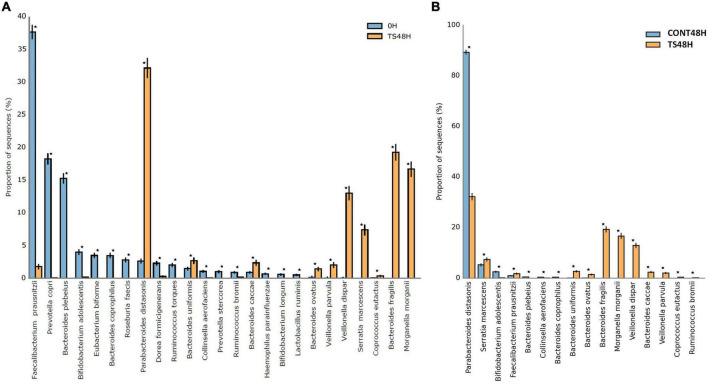
Statistical differences for the more abundant identified bacterial species in *Talitrus saltator*-added samples at 48 h of fermentation with respect to baseline **(A)** and with respect to negative control samples at 48 h of fermentation **(B)**. CONT, Negative controls; TS, *T. saltator*. *Indicates significant differences.

A total of 30 different bacterial species were identified in the current work. The results obtained from the statistical analysis comparing baseline samples with those obtained after 48 h of fermentation of *T. saltator* added samples showed significant differences for 25 bacterial species (Figures 3A,B). For 15 species (*Faecalibacterium prausnitzii*, *Prevotella copri*, *Bacteroides plebeius*, *Bifidobacterium adolescentis*, *Bacteroides coprophilus*, *Roseburia faecis*, *Dorea formicigenerans*, *Ruminococcus torques*, *Collinsela aerofaciens*, *Prevotella stercorea*, *Ruminococcus brommi*, *Haemophilus parainfluenzae*, *Bifidobacterium longum*, and *Lactobacillus ruminis*), the relative abundance was lower after 48 h of fermentation of *T. saltator* than at baseline, whereas 10 species were found in higher relative amounts after 48 h of fermentation of *T. saltator* than at baseline (*Parabacteroides distasonis*, *Bacteroides uniformis*, *Bacteroides caccae*, *Bacteroides ovatus*, *Veilonella parvula*, *Veillonela dispar*, *Serratia marcencens*, *Coprococcus autactus*, *Bacteroides fragilis*, and *Morganella morgani*). The higher relative abundance in *T. saltator*-added samples of genera belonging to the Bacteroidetes *phylum*, such as *Bacteroides* and *Parabacteroides*, shows that they could use polysaccharides from *T. saltator* as a carbon source, as they possess a wide range of genes encoding enzymes that allow the degradation of several polysaccharides (20). However, the higher relative abundance in *T. saltator*-added samples of some Proteobacteria, such as *M. morganii* or *S. marcencens*, is controversial because they are opportunistic pathogens (45, 46).

Comparing samples obtained after 48 h of fermentation of *T. saltator* with respect to negative controls, significant differences were found for a total of 16 bacterial species. In 11 species, relative proportions were higher after 48 h of fermentation of *T. saltator* (*B. fragilis*, *M. morganii*, *V. dispar*, *S. marcescens*, *B. uniformis*, *B. caccae*, *V. parvula*, *F. prausnitzii*, *B. ovatus*, and *C. autactus*), whereas in 5 cases, negative controls showed higher relative counts than *T. saltator*-added samples (*P. distasonis*, *B. adolescentis*, *C. aerofaciens*, *B. coprophilus*, and *B. pleveius*).

**FIGURE 4:**
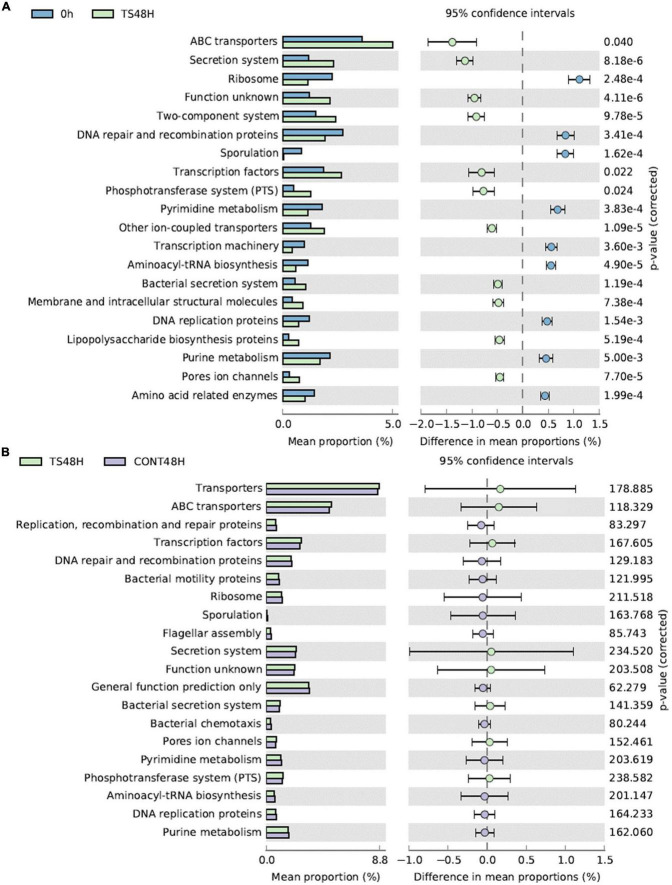
Main metabolic functions comparing *Talitrus saltator* added samples at 48 h of fermentation with respect to baseline **(A)** and with respect to negative control samples at 48 h **(B)**. CONT, Negative controls; TS, *T. saltator*.

Comparison of the 20 most frequent metabolic pathways was obtained with PICRUSt (Figure 4). At 0 h, the most frequent metabolic pathways were related to ABC transporters, transcription factors, the two-component system and the secretion system. These metabolic pathways remained the most frequent after 48 h of fermentation of *T. saltator* samples. However, interestingly, all metabolic pathways compared showed significant differences between baseline samples and samples obtained after 48 h of fermentation of *T. saltator*-added samples. For 11 cases, gut bacterial enrichment metabolic pathways were found after 48 h of fermentation of *T. saltator*-added samples with respect to baseline, and in 9 cases, a decrease in the metabolic pathways was found. Comparing *T. saltator*-added samples with negative controls, no significant difference in metabolic pathways was found.

## Conclusion

According to the results obtained from the nutritional composition, *T. saltator* meets the criteria to use the nutritional claims “high protein content,” “low fat content,” “low sugar content” and “source of fiber.” The toxic heavy metal contents were lower than the maximum levels legally established whereas some essential heavy metals, such as Zn, Cu and Fe double the minimum daily requirements. For the first time, *T. saltator* has also evaluated in terms of its effect on colonic human microbiota in an *in vitro* assay. *T. saltator* showed to exert some beneficial effects. as the maintaining of beneficial genera such as *Bifidobacterium* or *Lactobacillus* during the fermentation process, no increase Firmicutes in *phylum*, a decrease in the Firmicutes/Bacteroidetes ratio, and stimulation of 11 metabolic pathways with respect to baseline. Unusual in a high protein content-food. Thus, α-diversity (which is common in *in vitro* studies) decreased during the fermentation process and an increase in the Proteobacteria *phylum* was observed in a similar way to negative controls. The reported beneficial effects could be due to the chitin and complex carbohydrate content whereas those detrimental could be due to the low protein content. However, more studies are necessary to confirm these data, especially in the context of a whole diet.

## Data availability statement

The data presented in this study are deposited in the NCBI short read archive repository, accession number: PRJNA884699. https://www.ncbi.nlm.nih.gov/bioproject/884699.

## Author contributions

All authors listed have made a substantial, direct, and intellectual contribution to the work, and approved it for publication.

## Footnotes

1
https://usegalaxy.eu/
